# Exploring lifestyle management in polycystic ovary syndrome through implementation frameworks

**DOI:** 10.1113/EP092880

**Published:** 2026-08-14

**Authors:** Margaret McGowan, Stephanie Cowan, Chau Thien Tay, Lisa J. Moran, Ashley H. Ng

**Affiliations:** ^1^ Monash Centre for Health Research and Implementation, School of Public Health and Preventive Medicine Monash University Clayton Victoria Australia

**Keywords:** implementation, lifestyle management, polycystic ovary syndrome, polyendocrine metabolic ovarian syndrome

## Abstract

Polyendocrine metabolic ovarian syndrome [PMOS; previously known as polycystic ovary syndrome (PCOS)] is an endocrine condition affecting 10%–13% of women of reproductive age, with cardiometabolic, reproductive and psychological features. The PMOS guidelines have established recommendations for its management, which can include pharmacological, lifestyle and/or traditional medicines. The aim of this narrative review is to explore the pathophysiology, treatment recommendations and current evidence for lifestyle management for those with PMOS. Using the consolidated framework for implementation research (CFIR) and capability, opportunity, motivation and behaviour (COM‐B) frameworks, this review identifies barriers and facilitators to implement lifestyle recommendations for PMOS, followed by strategies to support improvement in lifestyle management. There is limited high‐quality evidence to support specific physical activity and dietary recommendations for those with PMOS. Underlying physiological mechanisms driving PMOS can impact energy expenditure, metabolism, appetite and satiety hormones, acting as barriers for weight and lifestyle‐management strategies. Qualitative research suggests that implementation of lifestyle‐management strategies from the PMOS guidelines has not been adopted. From a psychosocial perspective, those with PMOS often report that their health needs are not being met by health professionals. This might be related to a disproportionate focus on weight‐focused care, inadequate lifestyle‐management education for health professionals and inadequate support for people with PMOS to achieve lifestyle management. Working in partnership with those who have lived experience is crucial to translate the PMOS guidelines to improve health professional education, resources and adaption of lifestyle‐management programmes to meet the needs of those with PMOS better.

## Abstract (highlights)

- **What is the topic of this review?**
  This narrative review focuses on the barriers and facilitators on lifestyle management in polyendocrine metabolic ovarian syndrome [PMOS; previously known as polycystic ovary syndrome (PCOS)] across the systems, organisational and individual levels.
- **What advances does it highlight?**
  Key advances highlighted by this review include identification of physiological barriers unique to PMOS, such as altered metabolism, appetite hormones, sleep or fatigue, systemic gaps in health professional education and funding structures, and advocating for co‐designed, condition‐specific resources, adapted community programmes and evidence‐based mHealth tools, such as the AskPMOS app, to translate guideline recommendations into practice better.

## 1 INTRODUCTION

Polyendocrine metabolic ovarian syndrome [PMOS; previously known as polycystic ovary syndrome (PCOS)] is an endocrine condition affecting 10%–13% of women of reproductive age (Teede et al., 2023a, 2023b, 2023c, 2023d). Despite common misconceptions, PMOS does not include only reproductive features, such as irregular menstruation and infertility. Rather, there is also a range of cardiometabolic features (such as increased risk for type 2 diabetes and cardiovascular disease) and psychological features (such as increased prevalence of anxiety and depression) (Damone et al., 2019; Teede et al., 2018a), which have been increasingly recognised. PMOS is diagnosed when two of the following three symptoms are present: clinical or biochemical hyperandrogenism, an irregular menstrual cycle and/or polycystic ovarian morphology or elevated anti‐Müllerian hormone (AMH) levels (Teede et al., 2023a, 2023b, 2023c, 2023d). Symptoms of PMOS vary between individuals and can include hirsutism (excessive hair growth in all regions of the body, including the pubic area, legs, stomach and face), female pattern hair loss, acne, oily skin, acanthosis nigricans, low self‐esteem, disordered eating behaviours, sleep apnoea, excess weight gain and difficulty in losing weight. PMOS is therefore a complex condition that impacts the entire body, meaning that multifaceted treatment is required to address all unique concerns faced by someone with PMOS.

Management strategies for PMOS vary depending on the person's symptoms and treatment preferences, which can include pharmacological, lifestyle, traditional, complementary and integrative medicine, or a combination of all strategies (Teede et al., 2023a, 2023b, 2023c, 2023d). Lifestyle management is the recommended first line of treatment for PMOS, as highlighted in the 2023 International Evidence‐based Guideline for the Assessment and Management of Polycystic Ovary Syndrome (Teede et al., 2023a, 2023b, 2023c, 2023d). Lifestyle strategies are both effective in managing the array of symptoms experienced by people with PMOS and highly favoured (Al et al., 2021; Atkinson et al., 2021; Avery & Braunack‐Mayer, 2007; Copp et al., 2022; Douglas et al., 2021; Hajivandi et al., 2021; Hillman et al., 2020; Humphreys & Costarelli, 2008; Ismayilova & Yaya, 2022; Jeanes et al., 2009; Lim et al., 2021; Love et al., 2016; Pena et al., 2022; Pirotta et al., 2021; Rajkumar et al., 2022; Saslow & Aikens, 2020; Soucie et al., 2021; Tay et al., 2021; Thomson et al., 2016; Weiss & Bulmer, 2011; Williams et al., 2016). Similar to the general population, it is well established that lifestyle management can improve insulin resistance in people with PMOS, which has a beneficial impact on related symptoms, such as weight gain (Teede et al., 2023a, 2023b, 2023c, 2023d). Insulin resistance underpins many of the symptoms experienced by those with PMOS, independent of body weight (Teede et al., 2007), which emphasises the importance of lifestyle‐management strategies as first‐line therapy.

Despite the clear need for lifestyle management in PMOS, either in conjunction with or without other treatment therapies, there is limited evidence supporting a specific lifestyle approach for PMOS management, such as a PMOS diet, macronutrient distribution, physical activity volume or exercise regimen, other than the recommendations for the general population (Teede et al., 2023a, 2023b, 2023c, 2023d). However, the application of general lifestyle advice often does not consider the multifaceted nature of PMOS, including the physiological barriers to weight management (Nguo et al., 2024) and the psychosocial needs and experience of people with PMOS, such as poorer mental health (Brutocao et al., 2018).

This narrative review explores the current understanding of PMOS pathophysiology, management options, barriers and facilitators to lifestyle management and explores possible areas for future research through the lens of implementation and behavioural research frameworks.

## 2 PATHOPHYSIOLOGY AND AETIOLOGY

**FIGURE 1:**
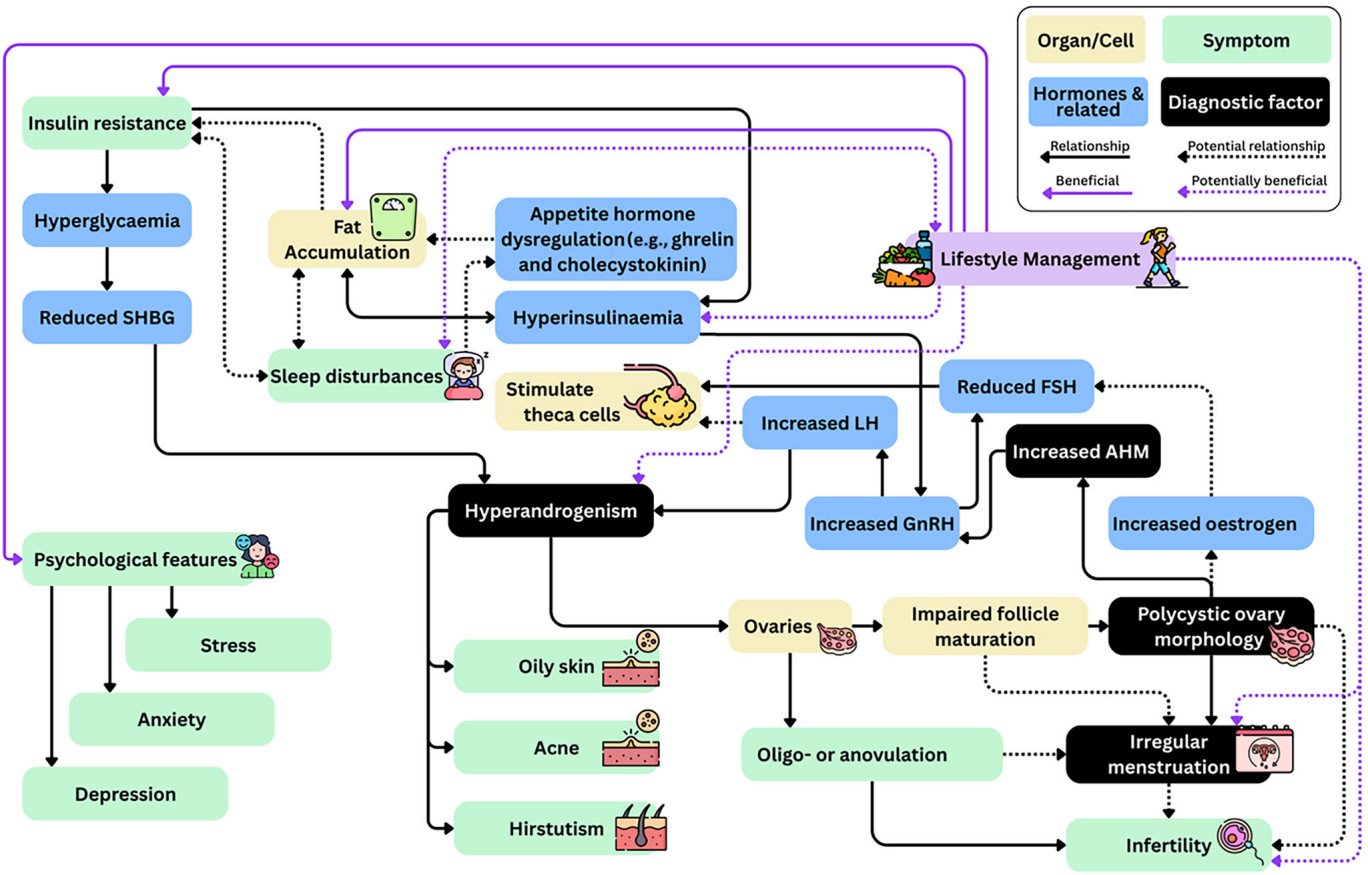
Clinical and pathophysiological factors in polyendocrine metabolic ovarian syndrome. Abbreviations: FSH, follicle stimulating hormone; GnRH, gonadotrophin‐releasing hormone; LH, luteinizing hormone; SHBG, sex hormone‐binding globulin.

The pathophysiology of PMOS is complex. Although its specific aetiology remains unclear, it relates to abnormalities across gonadotrophin function, hyperandrogenism and/or insulin resistance (Figure 1) (Stener‐Victorin et al., 2024).

In PMOS, gonadotrophin‐releasing hormone pulsatility is often elevated and increases the luteinizing hormone to follicle‐stimulating hormone ratio (Stener‐Victorin et al., 2010). As a result, luteinizing hormone stimulates theca cells to increase androgen production, resulting in hyperandrogenism (Stener‐Victorin et al., 2010). Hyperandrogenism can be present either biochemically or clinically (Teede et al., 2023a, 2023b, 2023c, 2023d). Biochemical hyperandrogenism refers to elevated serum androgen production (Legro et al., 1998), which can manifest as clinical hyperandrogenism features, including hirsutism, acne, female pattern hair loss and oily skin (Joham et al., 2022). However, biochemical hyperandrogenism does not need to be present for the features to occur (Escobar‐Morreale et al., 2012). Increased androgen production causes ovulatory disruptions, resulting in oligo‐/anovulation and leading to the classic polycystic ovary morphology (Escobar‐Morreale et al., 2012).

Intrinsic mechanisms in PMOS that drive insulin resistance include increased serine phosphorylation of insulin receptors, which impairs insulin signalling, reducing insulin sensitivity in muscle fibres (Dunaif et al., 1995), and impaired insulin‐stimulated glucose uptake in adipocytes (Ezeh et al., 2019, 2020). Insulin resistance can result in hyperinsulinaemia, which can be associated with weight gain, suppressing lipolysis and promoting lipogenesis in adipocytes (Thomas et al., 1979), with adiposity likely to exacerbate hyperinsulinaemia (Azziz et al., 2005). This highlights hyperinsulinaemia as one potential mechanism contributing to the causal and bidirectional relationship between PMOS and obesity (Joham et al., 2022). This relationship is complicated further by the observation that people with PMOS have a higher rate of longitudinal weight gain (Teede et al., 2013) and prevalence of weight gain and obesity (Lim et al., 2012; Teede et al., 2013) in comparison to people without PMOS, which can further worsen the features of PMOS (Lim et al., 2013). Furthermore, insulin resistance and the resulting hyperinsulinaemia reduce production of hepatic sex hormone‐binding globulin, which contributes to increased free circulating testosterone (Baillargeon & Carpentier, 2007). Hyperandrogenism might have a bidirectional relationship to insulin, increasing its release and contributing to hyperinsulinaemia.

People with PMOS are more likely to have mental health concerns, such as higher depression (Yin et al., 2021), higher anxiety (Yin et al., 2021), lower quality of life (Yin et al., 2021) and a higher prevalence of binge‐eating disorder and bulimia nervosa (Cooney et al., 2024). The mechanistic link between PMOS and poor mental health is not well understood. Direct pathophysiological contributions include the hypothesised role of hyperandrogenism in the increase of anxiety and depressive symptoms through androgen receptor activity and hypothalamic–pituitary–adrenal axis dysregulation (Stener‐Victorin et al., 2019). Indirectly, the psychosocial complexities associated with PMOS, including hirsutism‐related stigma, poor body image (Bazarganipour et al., 2014; Borghi et al., 2018; Kaczmarek et al., 2016) and concerns about body weight (Barry et al., 2011; Jones et al., 2008), are reported to be key contributors to poor mental health. These pathways are likely to be interacting and mutually reinforcing, although the mechanistic evidence still remains unclear.

The pathophysiology of PMOS is complex across multiple systems of the human body. Those with PMOS often experience a range of symptoms, which may present uniquely. Furthermore, prioritisation of symptomology can differ from person to person, based on life stage and symptom impact. Therefore, management should reflect these complexities and individual priorities. The complex interplay of several metabolic and reproductive hormones in PMOS is illustrated in Figure 1.

## 3 SYMPTOM MANAGEMENT FROM A LIFESTYLE PERSPECTIVE

There are different approaches to PMOS management, depending on symptoms and individual priorities, including pharmacological, lifestyle, traditional, complementary and integrative medicine, or a combination (Teede et al., 2023a, 2023b, 2023c, 2023d). The current PMOS guidelines define lifestyle interventions as those designed to improve dietary intake or physical activity through appropriate behavioural support (e.g., goal setting, monitoring) (Teede et al., 2023a, 2023b, 2023c, 2023d). These management strategies can be used either in isolation or in conjunction with each other.

### 3.1 Hyperandrogenism

Biochemical hyperandrogenism is a key pathophysiological and diagnostic feature in PMOS. Clinical hyperandrogenism symptoms include excess hair growth on the body and face (hirsutism), acne and female pattern hair loss (Teede et al., 2023a, 2023b, 2023c, 2023d). Lifestyle interventions have shown improvements in biochemical and clinical hyperandrogenism. A systematic review and meta‐analysis of 33 studies found that exercise‐only interventions showed a greater improvement in the free androgen index compared with diet alone (Patten et al., 2020). Although resistance training showed greater effects on lowering the free androgen index compared with aerobic exercise (Patten et al., 2020), a combined diet and exercise intervention had the greatest effect on the free androgen index (Kim & Lee, 2022; Patten et al., 2020). Additionally, a meta‐analysis of four studies found that lifestyle‐management strategies reduced hirsutism (Lim, Hutchison et al., 2019).

The PMOS guidelines support the use of the combined oral contraceptive pill, anti‐androgens and cosmetic therapy (e.g., waxing, shaving, mechanical laser and light therapies) to manage hirsutism (Teede et al., 2023a, 2023b, 2023c, 2023d). Inositol may be considered as an alternative therapy for hyperandrogenism, given its limited potential for harm, although this should be based on individual preference (Teede et al., 2023a, 2023b, 2023c, 2023d). When compared with lifestyle interventions, the combined oral contraceptive pill showed no differences in effect on the free androgen index and a greater effect on lower total free testosterone (Forslund et al., 2023). Although some research studies and systematic reviews have reported reductions in clinical hyperandrogenism after lifestyle interventions (Lim, Hutchison et al., 2019; Patten et al., 2020), these are generally of lesser effect compared with pharmacological therapies and might be of limited clinical relevance. The research, although limited, demonstrates the potential for lifestyle adoptions, such as healthy eating patterns and/or physical activity, to improve elements of hyperandrogenism.

### 3.2 Reproductive irregularities

Reproductive irregularities, including menstrual disruption and infertility, are major concerns for some people with PMOS (Teede et al., 2023a, 2023b, 2023c, 2023d). The combined oral contraceptive pill is often recommended for people with PMOS to regulate their menstrual cycle (Teede et al., 2023a, 2023b, 2023c, 2023d). For those wishing to conceive, letrozole is offered as a first‐line therapy to induce ovulation, followed by clomiphene citrate and metformin (either together or standalone) and gonadotrophins (Teede et al., 2023a, 2023b, 2023c, 2023d). Lifestyle modification has been shown to improve menstrual cyclicity (Arentz et al., 2017; Kim & Lee, 2022; Thomson et al., 2008; Turan et al., 2015) and to improve menstrual cycles and ovulation and reduce ovarian follicles compared with baseline levels (Kim & Lee, 2022; Shang et al., 2021). Specifically, a low‐calorie diet or a Mediterranean diet with or without energy restriction have demonstrated improvements in pregnancy and menstrual cycle regularity after 12 months (Shang et al., 2021). Exercise‐alone interventions showed mixed results, some supporting improvements in menstrual cyclicity (Al‐eisa et al., 2017; Vigorito et al., 2007), others showing no difference in luteinizing hormone and follicle‐stimulating hormone levels (dos Santos et al., 2020; Kite et al., 2019).

For fertility outcomes, dietary modification alone was associated with increased pregnancy rates, specifically a non‐energy‐restricted Mediterranean diet or a low‐carbohydrate diet for >3 months (Shang et al., 2021). A meta‐analysis of 18 studies reported a higher rate of live births, pregnancies and natural conception in physical activity interventions compared with controls. Additionally, there were no significant differences in outcomes when fertility treatments were compared with a physical activity intervention (Mena et al., 2019). Although there is limited research and heterogeneity in PMOS research for reproductive outcomes, it is likely that people with PMOS would benefit from meeting healthy lifestyle recommendations to improve menstrual regularity, ovulation and fertility outcomes.

### 3.3 Cardiometabolic features

Cardiometabolic features can be managed pharmacologically with metformin, with limited evidence supporting a combination of metformin and the combined oral contraceptive pill in those with a body mass index (BMI) ≤  30 kg/m^2^. However, this might potentially be beneficial in those with a BMI > 30 kg/m^2^ (Teede et al., 2023a, 2023b, 2023c, 2023d), impaired glucose tolerance or in high‐risk ethnic groups. Owing to the high prevalence of gastrointestinal side effects from metformin (Nabrdalik et al., 2022), it is also important to consider the cardiometabolic impacts of lifestyle interventions in the absence of pharmacotherapy. The improvement of metabolic features, such as reduction in body mass, and improvements in lipid profiles and fasting insulin following lifestyle intervention is well supported by literature (Kim & Lee, 2022; Nabrdalik et al., 2022; Naderpoor et al., 2015; Patten et al. 2020, 2020). The greatest improvements in metabolic features are seen in combined diet and exercise interventions (Kim & Lee, 2022), although no optimal dose has yet been determined (Patten et al., 2020). Furthermore, there were no differences in the majority of cardiometabolic outcomes (oral glucose tolerance test, insulin, fasting insulin, insulin sensitivity index, visceral and total adipose tissue, waist circumference, waist‐to‐hip ratio, fasting glucose, glucose area under the curve, lipids, blood pressure, BMI, fasting glucose, insulin area under the curve and glucose area under the curve) in a meta‐analysis of 12 studies comparing lifestyle interventions with or without metformin after 6 months (Naderpoor et al., 2015).

### 3.4 Weight

Excess adipose tissue contributes to extrinsic insulin resistance in PMOS (Stepto et al., 2013), as in the general population (Jiang et al., 2020) with a high BMI, therefore further exacerbating reproductive, metabolic and psychological features of PMOS (Lim et al., 2013). Weight management, defined as weight loss, weight maintenance and prevention of weight gain, is an important management strategy in PMOS for mitigating clinical features and future chronic disease risk (Teede et al., 2023a, 2023b, 2023c, 2023d). With regard to weight loss, there are a range of approaches to assist with this, including anti‐obesity medications, surgical interventions, meal replacement supplements and lifestyle management (Teede et al., 2023a, 2023b, 2023c, 2023d). Anti‐obesity medications, such as liraglutide or semaglutide, both glucagon‐like peptide‐1 receptor agonists, and orlistat or surgical interventions, such as bariatric surgeries, are associated with larger weight reductions than those achieved through lifestyle management alone (Goldberg et al., 2024). It is crucial that these strategies are instigated and sustained in conjunction with a healthy lifestyle (diet and physical activity) to improve long‐term outcomes. Women with PMOS gain, on average, 0.26 kg more per year in comparison to those without PMOS, as shown in longitudinal cohort studies (*n* = 14 247; over 19 years). However, significantly less weight gain is observed in those with healthier lifestyles (e.g., reduced sitting time, lower glycaemic index diet and increased physical activity) (Awoke et al., 2022). This suggests that small and sustainable lifestyle changes could assist with prevention of weight gain PMOS. Additionally, cognitive behavioural therapy has been shown to improve several outcomes in PMOS, including weight (Tang et al., 2022).

### 3.5 Mental health

It is well known that a healthy lifestyle can improve mental health symptoms, such as anxiety and depression, in the general population (Carraça et al., 2021; Grajek et al., 2022; Opie et al., 2015; Rebar et al., 2015; Zhang & Chen, 2019). The same principle applies to those with PMOS (Banting et al., 2014; Butt et al., 2023). Performing exercises over a range of intensities, doses, types and durations significantly improves multiple domains of the PMOS quality of life survey and the Medical Outcomes Study Short Form 36 health status questionnaire (Patten et al., 2021). Improvements in anxiety and depressive symptoms, measured by Depression Anxiety Stress Scale‐21, Hospital Anxiety and Depression Scale and Centre for Epidemiological Studies Depression Scale, have also been observed after aerobic or resistance‐based physical activity interventions (Patten et al., 2021). This demonstrates that participation in physical activity can be beneficial to those with PMOS, whether experiencing mental health symptoms or not, similar to the general population (Carraça et al., 2021; Rebar et al., 2015; Zhang & Chen, 2019).

Targeting specific symptoms or features present in people with PMOS might be the first step of personalised lifestyle management of PMOS. Although the 2018 PMOS guidelines supported phenotypes for PMOS (Teede et al., 2018b), phenotyping was later removed in the 2023 PMOS guidelines (Teede et al., 2023a, 2023b, 2023c, 2023d). Emerging research in PMOS has identified potential subtypes of POCS categorised by reproductive, cardiometabolic or background features (Van et al., 2024), and longitudinal research in Brazil might support this hypothesis (Soares Jr. et al., 2023).

## 4 BARRIERS AND ENABLERS TO LIFESTYLE‐MANAGEMENT STRATEGIES

In lieu of sufficient high‐quality evidence supporting any PMOS‐specific lifestyle recommendations (Teede et al., 2023a, 2023b, 2023c, 2023d), those with PMOS are recommended to follow guidelines for the general population in relationship to dietary, physical activity and behavioural management of lifestyle change (Department of Health and Aged Care, 2021; UK Chief Medical Officers', 2019; US Department of Health and Human Services, 2018). Only a small percentage of the general population currently meet the recommended lifestyle targets (Australian Bureau of Statistics, 2021a, 2021b; Centres for Disease Control and Prevention, 2020; Lee et al., 2022; NHS England, 2022), which is also true for those with PMOS for physical activity recommendations (Lin & Lujan, 2014) and healthy dietary guidelines (Lin & Lujan, 2014). Therefore, it is essential to understand an individual's ability to obtain, apply and maintain healthy lifestyle behaviours and implement the PMOS evidence‐based lifestyle recommendations from the guidelines.

**FIGURE 2:**
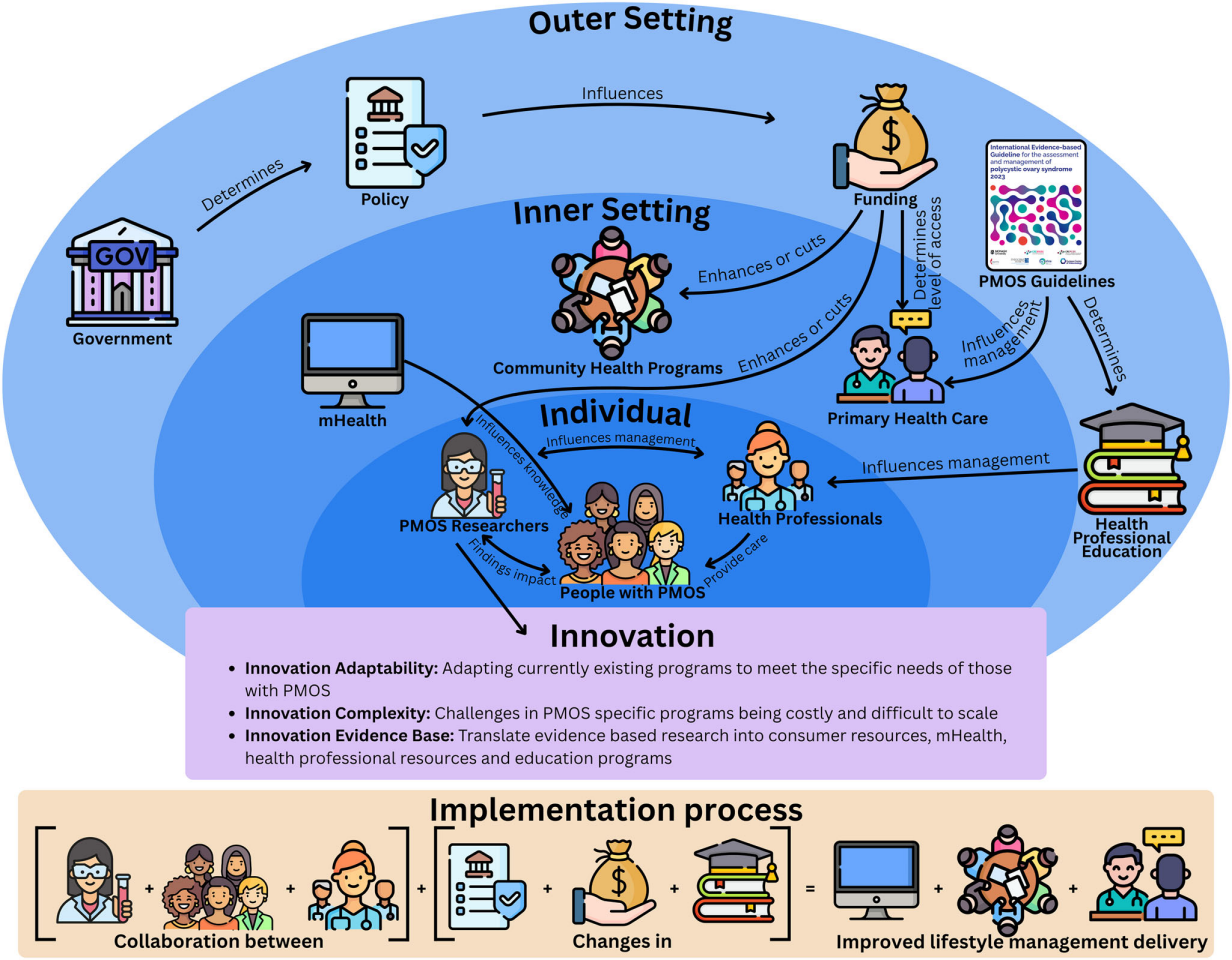
Summary of psychosocial barriers related to lifestyle management, mapped to the consolidated framework for implementation research framework. Abbreviation: PMOS, polyendocrine metabolic ovarian syndrome.

The consolidated framework for implementation research (CFIR) is one approach to assessing evidence‐based practice implementation. The CFIR identifies factors that might acts as barriers or facilitators, helping researchers and clinicians to navigate the challenges of implementing new practice or changes in practice (Damschroder et al., 2022). The five CFIR domains (individuals, inner setting, outer setting, innovation and the implementation process) are represented in Figure 2 as they apply to lifestyle management in PMOS. The outer setting captures all macro‐level influences, including PMOS guidelines, government policy and funding and health professional (HP) structures, which shape what care is available and how it is resourced (Damschroder et al., 2022). The inner setting refers to the health‐care environments where the innovation is delivered, such as primary care, community programmes and mHealth platforms, where lifestyle‐management strategies are delivered (Damschroder et al., 2022). Within the individual domain, people with PMOS, researchers and HPs supporting them bring their own capabilities, motivations and influences. Innovation can occur in any of the previously mentioned domains, including features of the intervention, such as policy changes, the structure and content of the intervention, the degree of adaptability or flexibility it allows, and the resources or tools required for its delivery (Damschroder et al., 2022). These domains are highly dynamic and interactive; for example, policy and funding decisions in the outer setting directly influence the resources and time available for HPs to consult with those with PMOS in the inner setting, subsequently shaping the intervention delivered to people with PMOS.

### 4.1 Outer setting

In the outer setting, there are three main influences that might impact the care received by those with PMOS: evidence‐based guidelines (the international PMOS guidelines; Teede et al., 2023a, 2023b, 2023c, 2023d), HP education, government funding and policy.

The 2023 International Evidence‐based Guideline for the Assessment and Management of Polycystic Ovary Syndrome is a cornerstone document that states the diagnostic criteria and best practice management strategies, including pharmacological, lifestyle and traditional medicines (Teede et al., 2023a, 2023b, 2023c, 2023d). The guidelines are informed by scientific evidence available at the time of publication and have been co‐designed to incorporate the lived experiences of those with PMOS (Teede et al., 2023a, 2023b, 2023c, 2023d). The PMOS guidelines outline the best practice management, including a person‐centred approach to care and coordinated support focusing on primary care and specialist clinicians (such as endocrinologists, gynaecologists and dermatologists) and allied health professionals (such as dietitians, exercise physiologists and psychologists), depending on an individual's needs (Teede et al., 2023a, 2023b, 2023c, 2023d). All care given and received should be based on this high‐level scientific evaluation (Teede et al., 2023a, 2023b, 2023c, 2023d).

Although the PMOS guidelines detail a structured approach to its management, the lifestyle strategies provided do not always meet the needs of those with PMOS. One study found that only 50% of those with PMOS received lifestyle‐management advice, and among those only 12% were satisfied with the care (Gibson‐helm et al., 2017). Furthermore, HPs and those with PMOS acknowledge that HP knowledge on PMOS lifestyle management is a limiting factor to effective delivery of education (McGowan et al., 2024). However, there is limited research investigating gaps in PMOS lifestyle‐management education for HPs. Qualitative research suggests that training relating to this is minimal during tertiary education, with further education on PMOS lifestyle management often being self‐directed (Gibson‐Helm et al., 2018b). Additionally, although those with PMOS want to receive PMOS‐specific support, including resources after consultations with HPs, there are limited readily available evidence‐based condition‐specific resources available (Gibson‐Helm et al., 2018b). Strengthening both HP education on how to deliver PMOS lifestyle‐management advice and evidence‐based resources can therefore help to build the capability of HPs to provide adequate care. However, capability alone is insufficient if structural barriers limit access to care.

To assist with multidisciplinary care delivery, the Australian government offers up to five subsidised allied health appointments (rebate of AUD$61.80 per appointment; Department of Health Disability and Ageing, 2025) per calendar year to support those living with chronic conditions (Australia Bureau of Statistics, 2025), including PMOS. For individuals with PMOS, the current allocation of five subsidised sessions per calendar year, combined with out‐of‐pocket costs created by gap fees, has been widely regarded as insufficient to meet their ongoing care needs (Chhour et al., 2022). Comparatively, in the USA, coverage of medical nutrition therapy (Medicare and Medicaid) varies depending on state, often not covering individuals with PMOS (Markus et al., 2026). Regardless of the geographical setting, those seeking care outside, beyond the government‐subsidised appointments, consider such services to be a major financial burden (Chhour et al., 2022). Furthermore, HPs who desire to provide longer consultations and in‐depth care are inhibited by reimbursement structures that do not compensate adequately for the time required to deliver best‐practice care (Arasu et al., 2019). Major systemic changes recommended in the outer setting to help improve financial access to lifestyle‐management support for PMOS include increasing the number of subsidised appointments per year, higher appointment cost coverage and appropriate reimbursement for HP time. Further contextual discovery needs to occur in each country, because each faces their own unique health‐care delivery and funding challenges.

When primary care is inaccessible for financial reasons, community‐based behaviour change programmes could be a potential cost‐effective solution for individuals and governments. However, the cost effectiveness of PMOS lifestyle‐management strategies has not been researched (Callander et al., 2025). In the general population, community‐based and behaviour change programmes have been found to be a cost‐effective way to prevent obesity (Lehnert et al., 2012). Adaptation of existing government‐funded lifestyle programmes with a focus on PMOS could be an additional outer setting change to assist with lifestyle management and to prevent the long‐term health implications of PMOS.

Finally, public health policy restricting social interactions might have impacted the opportunity for individuals with PMOS to access health‐care services and implement lifestyle changes. During the coronavirus disease 2019 (COVID‐19) restrictions, research from across the globe demonstrated substantial changes in lifestyle behaviours within the general population, including increased body weight (Bakaloudi et al., 2022), a decline in physical activity (Wunsch et al., 2022), and modified eating behaviours (González‐Monroy et al., 2021). Similar results have been observed in those with PMOS; however, the full impact of COVID‐19 restrictions and any longer‐term implications of these changes on those with PMOS are not understood (McGowan et al., 2023). This demonstrates the strong influence of public health policy on lifestyle habits and weight.

### 4.2 Inner setting

The inner setting provides the context for where lifestyle management for PMOS occurs. PMOS lifestyle‐management strategies are generally obtained in three settings: specialist and primary care, community‐based programmes and privately, online. Primary care refers to one‐to‐one consultations, which can occur in a public or private health setting with medical and allied HPs including general practitioners (GPs), endocrinologists, gynaecologists, dietitians, exercise physiologists and psychologists. Community programmes in the context of this paper are programmes that are low to no cost to individuals and run by government‐funded or not‐for‐profit organisations (such as Diabetes Victoria's *Life!* Program; Dunbar et al., 2014). Online can refer to social media, blog posts and other forms of self‐obtained information acquired through the Internet.

#### 4.2.1 Specialist and primary care

Primary care physicians, also known as GPs, are usually the first point of contact for people with PMOS. GPs provide initial management of PMOS, including support in diagnosis, education, lifestyle advice and pharmacological therapy, as required (Ee, 2023). Guided by the PMOS guidelines, GPs support individuals in developing a lifelong reproductive care plan and act as a facilitator of care, referring to multidisciplinary team members, including medical specialists (e.g., endocrinologists, gynaecologists, psychiatrists, dermatologists or fertility specialists) and allied health clinicians (e.g., psychologists, dietitians or exercise physiologists) where appropriate (Ee, 2023). A multidisciplinary approach to lifestyle management is highly valued (McGowan et al., 2024) and aligns with the PMOS guidelines (Teede et al., 2023a, 2023b, 2023c, 2023d). However, lifestyle‐management strategies as highlighted in the guidelines are currently not being achieved universally (McGowan et al., 2024), with allied HPs believing that they are underutilised in PMOS management (Moran et al., 2022). Improvements in referral pathways and multidisciplinary team structure and communication could assist in addressing this gap.

Within a multidisciplinary team, dietitians and exercise physiologists can provide specific, individualised lifestyle‐management strategies that align with the PMOS guidelines (Teede et al., 2023a, 2023b, 2023c, 2023d). Dietitians can provide medical nutrition therapy to assist with the management of PMOS (Nemchikova & Frontoni, 2022), while exercise physiologists can provide structured physical activity and exercise recommendations (Sabag et al., 2024). These allied HPs frequently use patient resources as a tool to reinforce verbal instructions and to assist with knowledge transfer and understanding of interventions (Tagtow & Amos, 2000). Individuals with PMOS desire these resources but often do not receive them (Ismayilova & Yaya, 2022; Saslow & Aikens, 2020). Resources have been shown to improve patient engagement in their own health (Carman et al., 2013), and HPs believe that increased engagement will improve patients’ knowledge, skill and confidence in managing their own health and health care (Carman et al., 2013; Gilliam, 2017). The co‐creation of freely accessible evidence‐based patient resources and PMOS education programmes for allied HPs should be explored to bridge the knowledge gap and to provide an innovative solution to the delivery of lifestyle management to those with PMOS.

The Monash Health PMOS clinic provides a blueprint for the application of the PMOS model of care in a specialist setting, as outlined in the PMOS guidelines (Tay et al., 2018). The clinic provides fully subsidised statewide multidisciplinary care to those with PMOS (Tay et al., 2021). The delivery of PMOS management from a medically trained and allied HP team can also be transferred to primary care settings (Tay et al., 2018). The PMOS clinic has been evaluated formally through extensive stakeholder engagement with medical and allied HPs and those with PMOS to guide service improvement (Tay et al., 2021). Through co‐design, this clinic has been tailored to meet the unique needs of individuals with PMOS, including access to an accredited practising dietitian (Tay et al., 2021). The Monash Health PMOS clinic is the only publicly funded PMOS‐specific clinic in Australia. To address the needs of those with PMOS, this model of care serves as a stellar example for national and global adoption (Tay et al., 2021).

#### 4.2.2 Community‐based programmes

It is well established that community‐based health‐prevention programmes can significantly reduce the risk of type 2 diabetes (Shirinzadeh et al., 2019; Shirvani et al., 2021), cardiovascular disease (Soltani et al., 2021) and obesity (Galani & Schneider, 2007) in the general population. Community‐based health programmes generally include a variety of lifestyle‐management strategies, including diet and physical activity (Galani & Schneider, 2007; Shirinzadeh et al., 2019; Shirvani et al., 2021; Soltani et al., 2021). Those with PMOS have a high risk of type 2 diabetes, cardiovascular disease and obesity and would therefore be likely to benefit from participating in community‐based health prevention programmes. However, those with PMOS have expressed a need for condition‐specific information over generalised health advice (McGowan et al., 2024). This highlights the importance of targeted, PMOS‐specific approaches to lifestyle‐management strategies, whether through dedicated programmes of adaptation of existing ones.

On a smaller scale, PMOS‐specific lifestyle programmes have been shown to improve some aspects of PMOS effectively, including improved emotional wellbeing (Jiskoot et al., 2020a) and weight reduction (Jiskoot et al., 2020b; Nikokavoura et al., 2015). However, these lifestyle programs can be intensive, costly (Lehnert et al., 2012) and difficult to scale in a real‐world setting. Conversely, existing community‐based lifestyle programmes might be lower intensity and affordable for existing health system partners. An alternative strategy is therefore to work with partners to adapt currently existing community‐based health prevention programmes to meet the specific needs of those with PMOS, such as Supplemental Nutrition Assistance Program (SNAP) and the Special Supplemental Nutrition Program for Women, Infants, and Children (WIC) in the USA (Hodges et al., 2024). In Australia, this includes Diabetes Victoria's *Life!* Program (Dunbar et al., 2014), a fully subsidised community‐based lifestyle modification programme targeting those at risk of type 2 diabetes. Leveraging these existing programmes to meet the needs of those with PMOS should be investigated further as a pathway for innovation.

#### 4.2.3 Mobile Health (mHealth)

When people with PMOS are unable to obtain adequate information through existing health system resources, such as specialist and primary care or community care, they often seek health information from other sources (Ismayilova & Yaya, 2022; Pirotta et al., 2021). This can include the Internet (Avery & Braunack‐Mayer, 2007; Copp et al., 2022; Douglas et al., 2021; Humphreys & Costarelli, 2008; Pirotta et al., 2021; Weiss & Bulmer, 2011; Williams et al., 2016), followed by social media when they are unable to find in‐depth knowledge through other online sources, such as websites (Gomula et al., 2024). However, social media posts are often non‐HP led (Horvath et al., 2024; Naroji et al., 2024) and provide low‐quality information and treatment options that have limited or no scientific evidence (Horvath et al., 2024). Additionally, a range of PMOS‐related apps also exist, which are often limited by the credibility of the information provided (Arabkermani et al., 2025). An exception to this is the evidence‐based AskPMOS app, which is affiliated with the PMOS guidelines and has the highest average mobile app rating scale, including a score of 4.57 out of 5 for information quality (Arabkermani et al., 2025).

The use of evidence‐based mHealth platforms for lifestyle modification in those with PMOS has shown improvements in weight loss and oocyte quality for those undergoing assisted reproductive technology treatment (Sang et al., 2022) and decreases in BMI, anxiety and depression and improvement in exercise and diet (Wang et al., 2022). Within the general population, improvements in diet and physical activity have also been shown when using mHealth platforms for lifestyle changes. It has also been reported that embedding HP interactions into mHealth platforms is more likely to lead to meeting lifestyle behaviour recommendations compared with no HP interactions (Jakob et al., 2022). In the absence of high‐quality PMOS information (Gomula et al., 2024) and readily and affordable accessible HPs, the utilisation of mHealth should be leveraged as an option for lifestyle‐management support. Existing high‐quality evidence‐based apps, such as the AskPMOS app, should be studied further to understand whether mHealth is an appropriate and feasible mode of delivery for lifestyle‐management strategies for those with PMOS.

### 4.3 Individual setting

To best describe challenges individuals might face when implementing change, the capability, opportunity, motivation and behaviour (COM‐B) framework (Michie et al., 2011), a behaviour change model, has been embedded within the CFIR. The COM‐B framework proposes that behavioural change occurs when an individual has the necessary psychological and physical capability, opportunity and motivation to perform the behaviour (Michie et al., 2011). To understand the challenges that people with PMOS face in meeting lifestyle recommendations, this section explores barriers and facilitators experienced from both the perspectives of those with PMOS and the HPs who support them.

#### 4.3.1 Physical capability: physiological barriers

**Energy expenditure and metabolism**. Early and limited research suggests altered energy expenditure and metabolism might be a barrier to weight management in those with PMOS (Nguo et al., 2024). Some research suggests that there is a decrease in basal metabolic rate (Georgopoulos et al., 2009) or postprandial thermogenesis (Robinson et al., 1992) in people with PMOS, although the research is inconsistent (Churchill et al., 2015; Robinson et al., 1992; Romualdi et al., 2019; Segal & Dunaif, 1990). Other research highlights a potential for the carbohydrate‐to‐fat oxidation ratio to be higher in those with PMOS in comparison to those without (Larsson et al., 2016), which is associated with weight gain in the general population (Seidell et al., 1992; Zurlo et al., 1990). Furthermore, those with PMOS might experience high metabolic inflexibility in comparison to the general population, making it more challenging to switch from lipid oxidation in fasting conditions to lipid availability in non‐fasting conditions. This has also been found in those with type 2 diabetes or experiencing obesity (Rimmer et al., 2020). These energy‐expenditure and metabolic differences might be associated with weight‐management challenges in those with PMOS (Nguo et al., 2018).

**Appetite**. Appetite hormones signal hunger and fullness to the body. Some research suggests that a range of appetite‐regulating hormones might be altered in those with PMOS. Ghrelin, an appetite‐stimulating hormone, was found, in a systematic review of 20 studies, to be lower in those with PMOS in comparison to those without (Gao et al., 2016). Those with PMOS who have a BMI ≥ 30 kg/m^2^ have a greater decrease in ghrelin beyond what would be expected in the presence of obesity (Pagotto et al., 2002). Furthermore, although ghrelin should decrease in the postprandial period, some research suggests that there is a blunted response in those with PMOS (Arusoglu et al., 2013; Zwirska‐korczala et al., 2008), which could potentially impact satiety. Cholecystokinin, a hormone responsible for inducing satiety and gut motility, has been reported to be lower in the postprandial period in those with PMOS (Lindén Hirschberg et al., 2004). Glucagon‐like peptide‐1, which helps to regulate blood glucose and appetite by stimulating insulin secretion, slowing digestion and increasing feelings of fullness, has been shown to be increased (Lin et al., 2015) or decreased (Aydin et al., 2014) during the fasting or postprandial period in those with PMOS.

In keeping with these findings relating to biochemical markers, self‐reported appetite dysfunction has also been reported in those with PMOS, with some feeling less satiated and hungrier after eating (Moran et al., 2004) and some studies reporting a higher energy intake and earlier return of hunger (Japur et al., 2019) in comparison to those without PMOS. Although some research supports the hypothesis of irregularities in energy expenditure, metabolism and appetite in those PMOS generating physiological barriers to lifestyle management, the evidence is mostly heterogeneous and inconsistent, making it challenging to draw clear conclusions around which specific mechanisms impact weight (Nguo et al., 2024).

**Fatigue and sleep**. In addition to potential alterations in energy homeostasis, there are also likely associations with sleep disorders impacting fatigue and perceived energy levels in PMOS. Fatigue is often a reported barrier to implementing lifestyle changes in both the general population (Deslippe et al., 2023) and those with PMOS (Lim, Smith et al., 2019; McGowan et al., 2024). Fatigue experienced by those with PMOS might be related to underlying physiological mechanisms. Fatigue and sleep are intrinsically linked, with fatigue (Hollinrake et al., 2007), poor sleep quality and sleep disorders (Azizi et al., 2020; Kahal et al., 2020; Thannickal et al., 2020) being reported at a higher frequency by those with PMOS in comparison to those without. People with PMOS experience obstructive sleep apnoea at a higher rate in comparison to those without PMOS (Abdul Jafar et al., 2025; Kahal et al., 2020) and are more likely to report sleep disturbances independent of obstructive sleep apnoea (Mo et al., 2019; Moran et al., 2015). The underlying mechanisms driving the difference in sleep disturbance between those with and without PMOS are unclear. However, they might include increased weight, psychological distress and upregulation of the hypothalamic–pituitary axis and/or increased sympathetic outflow owing to insulin resistance (Fernandez et al., 2018) and potentially high sex hormone‐binding globulin (Abdul Jafar et al., 2025).

#### 4.3.2 Psychological capability

The psychological capability to make lifestyle changes in PMOS can be impacted by the knowledge of HPs assisting with PMOS management and an individual's mental health. People with PMOS (McGowan et al., 2024) and HPs (Boyle et al., 2017; Carron et al., 2018; Chhour et al., 2022; Gibson‐helm et al., 2018a; Moran et al., 2022; Pathak, 2021; Wolf et al., 2018) acknowledge that inadequate lifestyle‐management education is a key barrier to receiving and delivering tailored advice. This leads to advice for lifestyle management being too general (Avery & Braunack‐Mayer, 2007; Bazarganipour et al., 2017; Djedjibegovic et al., 2020; Holbrey, 2013; Humphreys & Costarelli, 2008; Ismayilova & Yaya, 2022; Lin et al., 2018; Pathak, 2021; Pena et al., 2022; Snyder, 2006; Tomlinson et al., 2017) or weight‐loss focused (Avery & Braunack‐Mayer, 2007; Lim et al., 2021; Pirotta et al., 2021; Soucie et al., 2021; Tay et al., 2021; Tomlinson et al., 2017). HPs cite a lack of scientific research to inform PMOS lifestyle‐management strategy advice (Boyle et al., 2017; Chhour et al., 2022; Copp et al., 2020, 2022; Jeanes et al., 2009; Moran et al., 2022) and having limited time to deliver such education (Arasu et al., 2019; Chhour et al., 2022; Copp et al., 2022; Ko et al., 2016; Moran et al., 2022). As a result, there is a reduced ability for people with PMOS to obtain accurate information and consistent and evidence‐based lifestyle support, which negatively impacts their psychological capability to make changes. Furthermore, the mental health impacts of PMOS, including the burden of living with a chronic condition (Snyder, 2006; Thorpe et al., 2019), disordered eating (Atkinson et al., 2021; Ismayilova & Yaya, 2022; Love et al., 2016; Pirotta et al., 2021; Young et al., 2019), hyperandrogenism (Jones et al., 2008; Khomami et al., 2015; Pasch et al., 2016) and PMOS‐associated weight gain, low self‐esteem and poor body image (Love et al., 2016; Pirotta et al., 2021; Soucie et al., 2021; Thorpe et al., 2019; Williams et al., 2016) have been identified by people with PMOS as barriers to making dietary or physical activity changes (McGowan et al., 2024).

#### 4.3.3 Opportunity

Opportunity for behavioural change includes the social dynamics in an individual's life. People with PMOS have expressed that restrictive diets were difficult to maintain alongside social events (Atkinson et al., 2021; Pirotta et al., 2021; Thorpe et al., 2019; Weiss & Bulmer, 2011), social pressures around eating (Love et al., 2016; Young et al., 2019) and cultural norms (Carron et al., 2020). Alternatively, adequate emotional support, comfort and reassurance from peers with PMOS (McGowan et al., 2024), family (Banting et al., 2014; Rajkumar et al., 2022; Weiss & Bulmer, 2011; Young et al., 2019) and friends (Banting et al., 2014; Rajkumar et al., 2022; Weiss & Bulmer, 2011) have been perceived to assist in the application and maintenance of lifestyle recommendations. Although support and empathy from HPs was seen to enable lifestyle‐management changes, this has not met the expectations of those with PMOS (Atkinson et al., 2021; Ismayilova & Yaya, 2022; Soucie et al., 2021; Tay et al., 2021). Furthermore, societal perceptions of health and weight, including weight‐based assumptions (Humphreys & Costarelli, 2008; Pathak, 2021; Soucie et al., 2021; Tay et al., 2021; Tomlinson et al., 2017) within health systems, often inhibit social opportunities for change. For those in larger bodies, this manifested as weight loss often being the focus of lifestyle management, regardless of an individual's goals (Avery & Braunack‐Mayer, 2007; Lim et al., 2021; Pirotta et al., 2021; Soucie et al., 2021; Tay et al., 2021; Tomlinson et al., 2017). In comparison, those in smaller bodies are often perceived as ‘healthy’ and are not given the opportunity to receive lifestyle‐management advice (Arasu et al., 2019; Copp et al., 2022; Humphreys & Costarelli, 2008).

#### 4.3.4 Motivation

The extent to which people with PMOS are motivated to make and maintain lifestyle changes remains inconsistent across the literature. Some HPs perceive those with PMOS as having low motivation for sustained lifestyle changes (Arasu et al., 2019; Boyle et al., 2017; Chhour et al., 2022; Moran et al., 2022), whereas others have reported the opposite (Arentz et al., 2021; Atkinson et al., 2021; Copp et al., 2022; Lim et al., 2021; Pirotta et al., 2021; Rajkumar et al., 2022; Soucie et al., 2021; Williams et al., 2016; Young et al., 2019, 2020). People with PMOS reported being motivated by their family (Love et al., 2016), future good health (Pirotta et al., 2021; Tomlinson et al., 2017) and for symptom management [e.g. mental health (Atkinson et al., 2021; Love et al., 2016; Pirotta et al., 2021; Saslow & Aikens, 2020; Young et al., 2019), fertility (Holbrey, 2013; Ismayilova & Yaya, 2022; Love et al., 2016; Pena et al., 2022; Pirotta et al., 2021; Saslow & Aikens, 2020; Tomlinson et al., 2017), weight (Arentz et al., 2021; Love et al., 2016; Pena et al., 2022; Pirotta et al., 2021; Saslow & Aikens, 2020), regular menstruation (Pena et al., 2022; Saslow & Aikens, 2020), hirsutism (Pena et al., 2022; Saslow & Aikens, 2020), acne (Saslow & Aikens, 2020) and diabetes (Saslow & Aikens, 2020)] to make lifestyle changes. People with PMOS want condition‐specific lifestyle information from HPs that is accompanied by supportive materials summarising recommendations and guiding changes (McGowan et al., 2024). To ensure application and maintenance of lifestyle change, advice should be individualised, tailored to circumstances (Chhour et al., 2022; Copp et al., 2022; Moran et al., 2022), individual needs (Love et al., 2016), goals and motivations (Arasu et al., 2019; Copp et al., 2022; Love et al., 2016; Tay et al., 2021), culturally inclusive (Boyle et al., 2017; Carron et al., 2020; Copp et al., 2022; Ko et al., 2016; Pathak, 2021) and enjoyable and feasible (Boyle et al., 2017; Chhour et al., 2022).

## 5 CONCLUSION

PMOS is a complex endocrine condition with significant health implications across reproductive, cardiometabolic and psychosocial areas. Pharmacological and lifestyle changes are two of the management avenues that someone with PMOS can take. Lifestyle‐management strategies currently align with general population guidelines because there is insufficient evidence to support condition‐specific alternative approaches. However, generalised advice is not meeting the needs of those with PMOS or supporting them in achieving healthy lifestyle recommendations. When trying to obtain, apply and maintain lifestyle‐management strategies, those with PMOS face a range of physiological and psychosocial barriers, including changes in energy expenditure, metabolism, appetite, cost of health care, generalised management strategies and mental health challenges. Collectively, these barriers reflect a significant implementation gap. Despite established evidence‐based guidelines, more work needs to be done to translate lifestyle recommendations into consistent, accessible and person‐centred care for those with PMOS across the outer and inner settings and at the individual level.

Innovations in the outer settings, such as improved funding and access to health care, can assist in addressing some of the psychosocial barriers. However, changes in these settings can take time and are not always within immediate control beyond policy settings. In the interim, adaptations to already existing programmes, HP education and access to resources and mHealth platforms could assist in addressing some of the barriers identified, such as enhancing HP knowledge and consistency of information. To address the misalignment between current lifestyle‐management advice and the needs of those with PMOS, a person‐centred approach should be leveraged alongside the use of co‐design. Co‐designing education, resources, mHealth and adaptations to established programmes can help to bridge the gap between the wants and needs of those with PMOS and evidence‐based guidelines.

Closing this implementation gap requires a coordinated response. The most actionable priorities include the strengthening of PMOS‐specific lifestyle‐management training for HPs, increasing the number of government‐subsidised allied health appointments available to those with PMOS, and expanding the availability of freely accessible, co‐designed, evidence‐based resources for people with PMOS. Investment in the adaptation of existing community‐based lifestyle programmes to meet PMOS‐specific needs and further evaluation of mHealth platforms as scalable delivery mechanisms also represent high‐value opportunities. Larger‐scale policy advocacy could focus on the national replication of multidisciplinary PMOS clinic models, such as the Monash Health PMOS clinic. Future research should focus on addressing crucial gaps in the physiological understanding of PMOS and implementation strategies to assist with the obtaining, application and maintenance of lifestyle‐management strategies across a range of inner settings to meet the needs of a range of women with PMOS.

In summary, researchers must prioritise high‐quality implementation studies that evaluate real‐world adoption of lifestyle interventions across diverse populations and health‐care settings. Clinicians and allied HPs should be called upon to advocate for adequate resourcing and to embed co‐design principles into their practice. Policy‐makers must act to reform funding structures that currently limit equitable access to multidisciplinary PMOS care. Most importantly, those living with PMOS must be active partners in shaping the future of lifestyle‐management research, policy and practice.

## AUTHOR CONTRIBUTIONS

Margaret McGowan, Stephanie Cowan, Lisa J. Moran and Ashley H. Ng contributed to the conception and design of the work. Chau Thien Tay supported the interpretation of data for the work. All authors approved the final version of the manuscript, agree to be accountable for all aspects of the work in ensuring that questions related to the accuracy or integrity of any part of the work are appropriately investigated and resolved, and confirm that all persons designated as authors qualify for authorship and that all those who qualify for authorship are listed.

## CONFLICT OF INTEREST

The authors declare no conflicts of interest.

## FUNDING INFORMATION

The authors declare no funding for this work.

## GENERATIVE AI STATEMENT

Claude Sonnet 4.6 (Anthropic, 2025) was used to assist with drafting and language refinement. The authors retain full responsibility for the concepts, analyses and conclusions presented.
